# Prevalence, Severity, and Determinant Factors of Anemia among Pregnant Women in South Sudanese Refugees, Pugnido, Western Ethiopia

**DOI:** 10.1155/2016/9817358

**Published:** 2016-12-12

**Authors:** Aklilu Alemayehu, Lealem Gedefaw, Tilahun Yemane, Yaregal Asres

**Affiliations:** ^1^Department of Clinical Laboratory Science, Gambela Regional Health Bureau, Gambella, Ethiopia; ^2^Department of Medical Laboratory Science and Pathology, College of Health Sciences, Jimma University, Jimma, Ethiopia

## Abstract

*Background.* Anemia is one of the major health problems among refugee pregnant women in the world. Anemia among pregnant women is multifactorial and results in detrimental consequences on the mothers and infants. The aim of this study was to determine the prevalence, severity, and determinants of anemia among pregnant women in South Sudanese refugees, Pugnido western, Ethiopia.* Methods.* A facility-based cross-sectional study was conducted in Pugnido Administration Refugee and Returnee Affairs Health Center from April 15 to June 30, 2015. Demographic and related data were collected using questionnaire based interview. Complete blood count was done using CELL-DYN 1800 (Abbott USA). Blood smear and fecal specimen were examined for hemoparasite and intestinal parasite, respectively. Bivariate and multivariate logistic regression analyses were done using SPSS-Version 20.0.* Results.* The overall prevalence of anemia was 36.1%, from whom 2.3% had severe anemia. Being in third trimester, eating meat at most once a week, drinking tea immediately after meal at least once a day, having mid-upper arm circumference below 21 centimeters, and intestinal parasitic infection were identified as independent factors of anemia.* Conclusion.* More than one-third of pregnant women had anemia in this study. Intervention based strategies on identified determinant factors will be very important to combat anemia among the group.

## 1. Introduction

Anemia is a condition characterized by decrease in the number of red blood cells (RBCs) or their oxygen-carrying capacity to meet physiological needs of the body [1]. It varies by age, sex, and pregnancy condition. For example, anemia during pregnancy is defined as hemoglobin (Hb) concentration less than 11 g/dL [2–4]. Anemia is a public health problem: it affects one-quarter of the total population in the world [5]. Anemia among pregnant women is a serious continental issue in Africa. According to 1993–2005 global databases on anemia, World Health Organization (WHO) reported that 19.3 million (55.8%) pregnant women were anemic in Africa [5, 6]. Prevalence of anemia in African refugees ranged from 15.2% in Togo to 84.4% in Ethiopia [7–9].

Anemia among pregnant women is multifactorial. It is mainly resulted from nutritional deficiency particularly iron and folate deficiency, intestinal parasitic infection, malaria, and chronic illness. Gestational age, parity, consecutive birth interval, history of excess menstruation, and blood loss during pregnancy are among the most important obstetric and reproductive health related factors responsible for the occurrence of anemia during pregnancy [6, 10]. People living in refuges can suffer from different kinds of difficulties: nutritional deficiency is among the leading problems [11] and anemia is one of the nutritional problems commonly encountered [12]. Moreover, communicable diseases including intestinal parasitic infection and malaria can have a significant effect on the burden of anemia among pregnant women in refuges [9, 12, 13].

Anemia during pregnancy results in detrimental consequences on the mothers and infants [5]. The main consequences of anemia during pregnancy include higher risk of maternal and infant mortality, preterm delivery, and low birth weight [14–16]. Anemia is among the major causes of maternal deaths in refuges. From all maternal deaths reported in 2008 in Daddab refugee camp in Kenya, anemia was responsible for 55% of the total deaths [13]. Moreover, the negative outcomes of anemia on infants do not end at birth; rather it continues to affect their cognitive and physical development [4, 5].

Although anemia is largely preventable and treatable [10], it has been continued to be major problem among pregnant women in refuges. The management and prevention of anemia in pregnant women are essential for the wellbeing of not only the mother but also the fetus, newborns, and children. Strategies for prevention and control of anemia focused on its underline causes and determinant factors. But, review on epidemiological studies for the magnitude of anemia among pregnant women in Africa, specifically among pregnant women in refuges, reported varying prevalence and determinant factors. Moreover, there were limited data on the burden and determinant factors of anemia among pregnant women in refuges, Ethiopia. Hence, a particular data on the burden, severity, and determinant factors of anemia among pregnant women in refugee is desperately needed to combat anemia through targeted intervention strategies. Therefore, the aim of this study was to determine the prevalence, severity, and determinants of anemia among pregnant women in South Sudanese refugees, Pugnido, Western Ethiopia.

## 2. Materials and Method

### 2.1. Study Area and Population

The study was conducted at Pugnido Administration Refugee and Returnee Affairs (ARRA) Health Center in Pugnido Town, Western Ethiopia, from April 15 to June 30, 2015. Pugnido Town is the capital of Gog Woreda, located 885 km to the west of Addis Ababa [17]. Gog Woreda has three governmental and two ARRA health centers, 12 health posts, and three private clinics. The Woreda has one refugee camp near Pugnido Town inhabited by 54,597 refugees at the end of February 2015. The total number of pregnant women in the refugee camp was 3,200 in the year. The livelihood of almost all refugees was dependent on aid from different donors found in the region. Maize and wheat flour distributed by the donors and fish available from the local market were the main dietary options consumed in the refugee camp [18].

The required sample size was determined using single population proportion formula [19]. For the determination of sample size, 95% confidence interval, 5% margin of error, and 62.6% prevalence of anemia [20] were considered. Based on all the above considerations, the calculated sample size was 360. All Pugnido refugee camp pregnant women who attended antenatal care (ANC) clinic at Pugnido ARRA Health Center during data collection period were included in this study consecutively. Pregnant women who received blood transfusion four months prior to data collection and who were on treatment of anemia were excluded from the study.

### 2.2. Data Collection

Demographic, obstetric, clinical, and nutritional data were collected using structured and interviewer administered questionnaire. The questionnaire was adapted from Ethiopian Demographic and Health Survey (EDHS) 2011 and related literatures. The interview was done when the pregnant women came to the health center for ANC service, but after the assessment of eligibility and having their consent. It took a maximum of 35 minutes.

After the interview had been completed, two midwifery data collectors took middle upper arm circumference (MUAC) from each study participant. MUAC was measured at the midpoint between the tip of the shoulder and the tip of the elbow on left arm using four color tapes. A MUAC value less than 21 cm indicated malnutrition [21].

At the same day of questionnaire administration and anthropometric measurement, four milliliters of venous blood was collected from each study participant using ethylene diamine tetra acetic acid (EDTA) anticoagulated test tubes. Complete blood count was done using CELL-DYN 1800® (Abott Laboratories Diagnostics Division, USA) within 2 hours of sample collection in ARRA Health Center Laboratory. Pregnant women who had Hb concentration of 10–10.9 g/dL, 7–9.9 g/dL, and less than 7 g/dL were considered as mildly, moderately, and severely anemic, respectively [2]. Thick and thin blood films were prepared from EDTA anticoagulated blood sample and stained using 10% Giemsa stain for microscopic examination of hemoparasites. Moreover, stool specimens were collected from all study participants using clean, leak proof stool cups. Stool wet mount and formol-ether concentration techniques were performed for examination of intestinal parasites.

To ensure the quality of data, data collectors were trained for two consecutive days on data collection tool and anthropometric measurement techniques prior to commencement of the study. Questionnaire was pretested and translated to local languages, Nuer and Agnuwa. Pretest was done on 18 volunteer pregnant women in Itang refugee camp attending Itang Health Center prior to data collection. After the pretest had been completed, there were no errors found to be revised, but it helped us to determine the time required for interview. All laboratory activities were done using standard operating procedures. Laboratory reagents, quality control materials, and instruments were used strictly following manufacturers instruction. All reagents and quality control materials were checked for their expiry date. Assayed control materials, low, normal, and high were used for CELL-DYN 1800 to check its reliability.

### 2.3. Data Processing and Analysis

Data were coded, cleaned, and entered into EpiData version 3.1 and exported to SPSS-Version 20 (SPSS, Chicago, IL, USA) for analysis. Descriptive analysis was performed to summarize the sociodemographic, obstetric, parasitic infection, nutrition, and ANC service utilization related data. The association between anemia and each variable was analyzed using binary logistic regression analysis. Explanatory variables with *P* value ≤ 0.20 in binary logistic regression analysis were selected as candidates for multivariate logistic regression analysis to identify independent predictors of anemia. The *P* value ≤ 0.20 was used as a cutoff value in consideration of our sample size and number of variables [22]. *P* values, odds ratios, and 95% confidence intervals were used to present bivariate and multivariate logistic regression analysis. For all statistical tests, *P* value < 0.05 was considered as statistically significant.

### 2.4. Ethical Consideration

Ethical clearance was obtained from Jimma University College of Health Sciences Ethical Review Board. Support letters were obtained from the Gambela Regional Health Bureau and ARRA Regional Office. Agreement of study participants to participate in the study was ascertained by written informed consent. Informed consent for pregnant women who had less than 18 years of age was taken from each respective relative or partner. Moreover, an assent was taken from each study participant who had less than 18 years old. Confidentiality was ascertained by anonymization of the data that personal identifier was not disclosed beyond data collectors, supervisors, and investigator. Pregnant women who had anemia and/or parasitic infection were referred to their clinician at the ANC department for interventions.

## 3. Result

### 3.1. Sociodemographic and Obstetric Characteristics

Three hundred sixty pregnant women were enrolled in this study. The mean age of the study participants was 25.8 (±6.6) ranging from 16 to 42 years. Half of the study participants (*n* = 180) lack formal education, from whom 62.8% (*n* = 113) were unable to read and write. Pugnido refugee camp is mainly inhabited by Nuer population, 63.9% (*n* = 230) (Table 1). The mean gestational age of the study participants was 25.3 (±7.5) weeks, ranging from 10 to 41 weeks.

### 3.2. Nutrition and Clinical Characteristics

**Figure 1:**
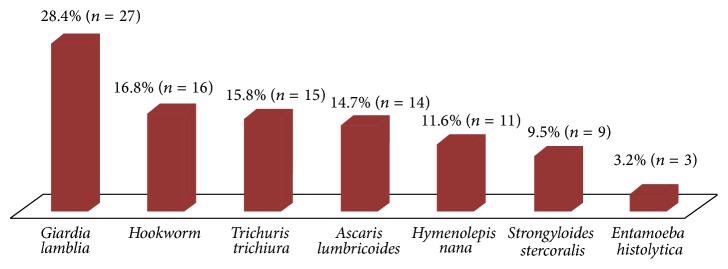
Proportion of different intestinal parasites among intestinal parasite infected pregnant women attending ANC clinic at Pugnido ARRA Health Center in Gambela, Western Ethiopia, April 15–June 30/2015 (*n* = 95).

Majority of study participants in this study, 89.2% (*n* = 321), consume meat at least ones during index pregnancy. Porridge was the staple food for 69.2% (*n* = 249) of study participants. Mean value of MUAC measurement in this study was 22.9 ± 2.4 cm, with the minimum and maximum of 16 cm and 30 cm, respectively. The majority of study participants, 76.7% (*n* = 276), attended ANC clinic for the first time and there was no pregnant woman on a fourth ANC visit (Table 1). Majority of study participants, 76.7% (*n* = 276), were visiting the ANC clinic for the first time and there was no pregnant woman on fourth ANC visit (Table 1). The prevalence of intestinal parasitic infestation was 26.4% (*n* = 95), from which* Giardia lamblia* 28.4% (*n* = 27) took the highest proportion (Figure 1). The prevalence of malaria infection in this study was 15.9% (*n* = 57). The most prevalent species was* plasmodium falciparum*, 82.5% (*n* = 47), followed by* plasmodium vivax* 14% (*n* = 8) and 3.5% (*n* = 2) mixed infection.

### 3.3. Prevalence and Severity of Anemia

**Figure 2:**
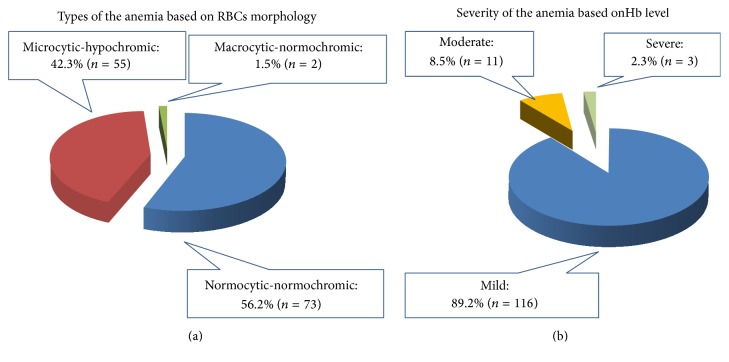
Morphological classification (a) and severity (b) of anemia among anemic pregnant women attending ANC clinic at Pugnido ARRA Health Center in Gambela, Western Ethiopia, April 15–June 30/2015 (*n* = 130).* Mild anemia*: hemoglobin concentration between 10 g/dL and 10.9 g/dL;* moderate anemia*: hemoglobin concentration between 7 g/dL and 9.9 g/dL;* severe anemia*: hemoglobin concentration less than 7 g/dL;* normocytic-normochromic anemia*: hemoglobin concentration less than 11 g/dL, MCV value between 80 fL and 100 fL and MCHC value between 32 g/dL and 36 g/dL;* microcytic-hypochromic anemia*: hemoglobin concentration less than 11 g/dL, MCV value less than 80 fL and MCHC value less than 32 g/dL;* macrocytic*: MCV value greater than 100 fL.

The mean Hb concentration of the study participants was 11.3 ± 1.5 g/dL, with the minimum and maximum value of 6.2 g/dL and 17.5 g/dL, respectively. The mean MCV, MCH, and MCHC values of the study participants were 85.3 fL, 27.2 pg, and 32.1 g/dL, respectively. The overall prevalence of anemia in this study was 36.1% (*n* = 130). The prevalence of mild, moderate, and severe anemia in this study was 32.2% (*n* = 116), 3.1% (*n* = 11), and 0.9% (*n* = 3), respectively. From all anemic study participants, 89.2% had mild anemia and 56.1% (*n* = 73) had normocytic-normochromic type of anemia (Figure 2). Binary logistic regression analysis indicated that educational status and malaria infection were associated with anemia (*P* value < 0.05) (Tables 2 and 3).

### 3.4. Independent Predictors of Anemia

Sixteen explanatory variables which had *P* value ≤ 0.20 in binary logistic regression analysis were analyzed by multivariate logistic regression analysis. Finally, being in third trimester of pregnancy (AOR = 3.12, 95% CI: 1.16–9.83), eating meat at most once a week (AOR = 2.00, 95% CI: 1.11–3.58), drinking of tea immediately after meal at least once a day (AOR = 3.01, 95% CI: 1.74–5.22), having mid-upper arm circumference below 21 centimeter (AOR = 3.90, 95% CI: 1.94–7.84), and intestinal parasitic infestation (AOR = 2.17, 95% CI: 1.20–3.91) were identified as independent predictors of anemia in this study (Table 4).

## 4. Discussion

Based on WHO cutoff values, anemia prevalence in this study indicated a moderate public health problem. Being in third trimester, drinking tea immediately after meal at least once a day, eating meat at most once a week, MUAC measurement less than 21 cm, and intestinal parasitic infestation were identified as independent predictors of anemia. The overall prevalence of anemia in this study, 36.1% (*n* = 130), was found consistent with the study finding in Palestinian refugees (38.6%) [23], South Sudanese refugee living in refugee camps of Uganda (36.3%) [8] and Malaysia (35.0%) [24]. Prevalence and trend of anemia saw a significant decline through time (2005–2011) in most regions of Ethiopia including Gambela [25]. But the magnitude of anemia among pregnant women in this study still continues to be public health problem which is an indicator of the need of coordinated effort to its prevention and control.

Our finding was lower than reports from WHO estimation (62.7%) [5], Saharawi refugees in Algeria (76.5%) [9], Afghan refugees in Pakistan (42.5%) [26], Rural India (74.8%) [27], Eastern Sudan (62.6%) [20], Niger Delta, Nigeria (66.7%) [28], and Gilgel Gibe Dam area in Ethiopia (53.4%) [29]. This might be due to methodological variation; for example, reports from Gilgel Gibe Dam area [29] and Saharawi refugees in Algeria [9] were community-based studies. Large sample size in Pakistan [26] and Eastern Sudan [20] studies might have a contribution for this variation. Unlike our study, exclusion of those in first trimester from the study done in Rural India [26] might also conceivably raise the prevalence.

The current study finding revealed higher prevalence of anemia compared to studies conducted in USA, Bhutanese refugee camp (28%) [30], East Anatolian Province, Turkey (27.1%) [12], Gondar, northwest Ethiopia (16.6%) [31], and Addis Ababa, Ethiopia (21.3%) [32]. This difference might be due to better socioeconomic status among pregnant women in Bhutanese refugees in USA [30], Turkey [12], and Gondar [31]. For example, a study done in Gonder reported that low monthly income was an independent predictors of anemia among pregnant women [31] while in this study majority of study participants had no source of income other than aid or donation. This might be an indication that economical empowerment or income generating for pregnant women in our study area might have a contribution to reduce the burden of anemia. Exclusion of study participants who had chronic illness and antepartum bleeding in the study done in Addis Ababa Ethiopia [32] might also have brought the difference. Higher number of pregnant women in third trimester and relatively higher prevalence of parasitic infection in our study might have increased the anemia burden.

Mild anemia was the common form of anemia in this study; 89.2% of anemic pregnant women had mild anemia. This finding is concordant with similar studies in Tikur Anbessa, Addis Ababa Ethiopia (80.9%) [32], Kakamega, Eastern Sudan (52.4%) [20], and Palestine (92.4%) [23]. The possible explanation for this finding could be the inclusion of pregnant women in the first trimester during which intensive hemodilution is not occurring and those received hematinics that help to prevent further fall in Hb concentration. Moderate anemia was reported as the major form among pregnant women from similar studies in Wolayta Sodo Town, Southern Ethiopia (60%) [33], Karnataka, India (50.4%) [34], and West Algeria (49.5%) [35]. The difference might be emanated from exclusion of pregnant women who were on hematinics [33] unlike the current study. Exclusion of pregnant women, who were in the first trimester in the Indian study, might have contribution for the observed difference. The other possible source of inconsistency with the study done in West Algeria might be involvement of all pregnant women from third trimester. The proportion of severe anemia among anemic respondents was 2.3%, which is almost similar to reports from Eastern Sudan (2.2%) [20] and Tikur Anbessa, Addis Ababa Ethiopia (1.19%) [32]. However, our finding was considerably lower than study in Rural India (18.9%) [27], excluding pregnant women in the first trimester, which could possibly justify the discrepancy.

Considering the morphological classification of anemia, normocytic-normochromic anemia (56.2%) was the major type in this study. This might be due to the fact that intestinal parasitic infection and antepartum bleeding might cause a fall in Hb concentration while keeping the red cell indices. Our finding was not consistent with results from studies done in Uyo University Teaching Hospital in Nigeria (66.5%) [36] and Krishna District [37], where microcytic-hypochromic anemia was most common. This inconsistency might have emanated from inclusion of pregnant women visiting the ANC clinic more than once, who had received iron/folate supplement in our study.

Sociodemographic factors can play a big role in determining anemia among pregnant woman. In this study, sociodemographic characteristics did not show any significant association with anemia. The possible explanation for this finding might be the similarity of the respondents as all of them were from the refugee camp. More importantly, almost all of the refugees utilize similar services provided by aid organizations, which might narrow the gap among the participants with respect to these variables. This condition continued to affect other characteristics in this study making them to show nearly communal tendency in predicting the anemia than individual level.

Obstetric characteristics of a pregnant woman play an important role in determining Hb status through increasing requirement for iron or depletion of its storage [3]. Despite this fact, our study did not show significant association with anemia. The only exception was gestational age: pregnant women in the third trimester were 3.12 (95% CI: 1.16–9.83; *P* = 0.014) times more likely to be anemic compared to those in the first trimester. This can be related to increased nutritional need for the rapidly growing fetus during third trimester. In line with our finding, third trimester was reported as independent predictors of anemia in Palestine [23], East Anatolian Province, Turkey [12], and Wolayta Sodo Town, Southern Ethiopia [33]. The current result was inconsistent with finding from Southeast Ethiopia [38] and Niger Delta Nigeria [28], where gestational age was not significant factor of anemia. The presence of relatively greater proportion of pregnant women in third trimester in the current study might cause the difference.

Parity and birth interval between successive births were reported as predictors of maternal anemia in many studies [23, 32, 33, 39] but not in this study. The possible reason for this difference might be attainment of relatively longer birth interval among our respondents compared to studies in Addis Ababa [32] and Southwest Ethiopia [39]. Larger sample size and relatively higher proportion of multiparous in Palestine [23] and multigravidae in Wolayta Sodo [33] might be the reason for the difference. History of abortion, prolonged menstruation, and bleeding during index pregnancy did not show significant association with anemia in this study. Contradicting result was reported from study done in Southeast Ethiopia [33], where history of heavy menstruation was reported as predictors of anemia. The prevalence of history of prolonged menstruation among pregnant women in our study (11.4%) was lower than in Southeast Ethiopia (30.2%) [39]; this might be the reason for discrepancy.

In this study anemia was significantly associated with intestinal parasitic infestation. Pregnant women who had intestinal parasitic infection were 2.17 (95% CI: 1.20–3.91; *P* = 0.010) times more likely to be anemic. This can be due to ability of those parasites to bring-about gastrointestinal blood loss which results in anemia. Malaria infection can cause anemia by triggering destruction of red cell [4]. The current study did not indicate any significant association between malaria infection and anemia while other studies [29, 40, 41] reported significant association between malaria infection and anemia. This might not be an indicator that malaria infection did not have an effect on anemia; rather the variation might be attributed to relatively lower malaria prevalence compared to studies in Kisumu, Western Kenya [40], and Enugu, Southeast Nigeria [42]. Application of community-based study design in Gilgel Gibe Dam area [29] and Sidama Zone, Southern Ethiopia [41], might be another reason for the difference.

Nutritional anemia develops if there is increased need accompanied by limited supply of the specific micronutrient [2, 4]. Inadequate consumption and improper dietary habit with respect to the necessary micronutrient during pregnancy can increase the risk of developing anemia. Pregnant women eating meat at most once per week were 2 (95% CI: 1.11–3.58; *P* = 0.020) times more likely to develop anemia compared with consumption frequency more than once per week in this study. In line with our finding, frequency of meat consumption was indicated as independent predictor of anemia among pregnant women from the studies done in West Arsi Zone, Ethiopia [43], and Fayoum Governorate, Egypt [44]. This might be due to the fact that meat is the best source of heme iron. But, study done in Addis Ababa, Ethiopia [32], reported absence of significant association between anemia and meat consumption. The discrepancy might be related to presence of greater number of participants, who consumed meat in Addis Ababa than the current study.

In this study, pregnant women who had MUAC measurement below 21 cm were 3.90 (95% CI: 1.94–7.84; *P* < 0.001) times more likely to be anemic compared to those who had at least 21 cm. This finding is supported by similar study from Westmoreland, Jamaica [45]. This can be explained by the fact that pregnant women suffering from malnutrition are more likely to be micronutrient deficient including iron, which indisputably leads to anemia. In the current study, pregnant women who took tea immediately after meal at least once a day increased the risk of being anemic by 3.01 (95% CI: 1.74–5.22; *P* < 0.001). This was concordant with the findings from West Arsi Zone, Ethiopia [43], and Fayoum Governorate, Egypt [44]. This might be due to the fact that tea contains a tannin molecule that inhibits the absorption of nonheme iron; it considerably affects the absorption if taken immediately after meal. This might be an indicator for advising pregnant women on nutritional habit which will be very important to prevent and control anemia.

In this study, we have tried to put the first effort on problem of previously untouched community: the refugee pregnant women in Ethiopia using primary data. But, our study design was cross-sectional; it was difficult to verify whether anemia preceded the predisposing factors or the vice versa. Moreover, micronutrients were not assessed due to logistic constraints.

## 5. Conclusion

More than one-third pregnant women had anemia in this study. According to WHO cutoff value, anemia was a moderate public health problem in our study area. Most of the identified predictors of anemia in this study were related to nutrition and nutritional habit which is directly or indirectly an indication of food insecurity among pregnant women attending ANC Clinic in HAARA Health Center in Pugnido refuge. Therefore, intervention based strategies on identified determinant factors specifically; prevention and control of intestinal parasitic infection and nutrition related problem solving strategies will be very important to combat anemia among the group. Moreover, another study focus on the rout courses of anemia including micronutrient among pregnant women might be very important.

## Figures and Tables

**Table 1:** Sociodemographic, obstetric, nutrition, and clinical characteristics of refugee pregnant women attending ANC clinic at Pugnido ARRA Health Center, Gambela, western Ethiopia, April 15–June 30/2015.

Variables	Freq. (%)
Age in years	
16–20	107 (29.7)
21–25	90 (25.0)
26–30	90 (25.0)
31–35	41 (11.4)
≥36	32 (8.9)
Ethnicity	
Agnuwa	130 (36.1)
Nuer	230 (63.9)
Occupation	
Students	85 (23.6)
Housewife	133 (36.9)
Employed	29 (8.1)
No specified job	113 (31.4)
Income availability^*∗*^	
Yes	73 (20.3)
No	287 (79.7)
Educational status	
Illiterate	113 (31.4)
Literate	247 (68.6)
Marital status	
Married	288 (80.0)
Unmarried^*∗∗*^	32 (20.0)
Duration of stay in refugee camp	
<18 months	148 (41.1)
≥18 months	212 (58.9)
Trimester	
First	34 (9.5)
Second	165 (45.8)
Third	161 (44.7)
Gravidity	
Primigravidae	78 (21.7)
Multigravidae	282 (78.3)
Parity	
Nulliparous	91 (25.3)
Primiparae	31 (8.6)
Multipara	238 (66.1)
Birth interval in months	
≤24	115 (48.3)
>24	123 (51.7)
Blood loss^*∗∗∗*^	
Yes	51 (14.2)
No	309 (85.8)
Prolonged menstruation^†^	
Yes	41 (11.4)
No	319 (88.6)
History of abortion	
Yes	64 (22.7)
No	218 (77.3)
Freq. of meat eating per week	
At most once	192 (53.3)
More than once	168 (46.7)
Freq. of vegetable eating per week	
At most once	141 (39.2)
More than once	219 (60.8)
Freq. of fruit eating per week	
At most once	234 (65.0)
More than once	126 (35.0)
Staple food	
Fish	44 (12.2)
Porridge	249 (69.2)
Injera	60 (16.7)
Other^††^	7 (1.9)
Freq. of drinking tea immediately after meal per day	
At least once	122 (33.9)
Less than once	238 (66.1)
Freq. of drinking coffee immediately after meal per day	
At least once	95 (26.4)
Less than once	265 (73.6)
MUAC	
<21 cm	68 (18.9)
≥21 cm	292 (81.1)
Number of ANC visits for current pregnancy	
1	276 (76.7)
≥2	84 (23.3)
Taken iron/folate supplement^†††^	
Yes	69 (19.2)
No	291 (80.8)
Place of previous delivery	
Health institution	123 (45.7)
Home	146 (54.3)
ANC follow-up in previous pregnancy	
Yes	119 (44.2)
No	150 (55.8)
History of malaria infection	
Yes	215 (59.7)
No	145 (40.3)
Positive for current malaria infection	
Yes	57 (15.8)
No	303 (84.2)
Positive for intestinal parasite infestation	
Yes	95 (26.4)
No	265 (73.6)

^*∗*^Any source of personal income other than aid or donation; ^*∗∗*^single, divorced, and widowed; ^*∗∗∗*^bleeding (hemorrhage) during index pregnancy; ^†^history of prolonged menstruating for longer than 7 days; Freq.: frequency; ^††^respondents using bread and packed food as their staple food; ^†††^taken iron/folate supplement during the index pregnancy; MUAC: mid-upper arm circumference; ANC: antenatal care.

**Table 2:** Sociodemographic and obstetric characteristics of the refugee pregnant women attending ANC clinic at Pugnido ARRA Health Center in Gambela, Western Ethiopia, April 15–June 30/2015.

Variables	Anemia		COR (95% CI)	*P* value
	Yes: *n* (%)	No: *n* (%)		
Age in years				
16–20	37 (34.6%)	70 (65.4%)	1.89 (0.85–4.20)	0.118^*∗*^
21–25	35 (38.9%)	55 (61.1%)	1.57 (0.69–3.54)	0.275
26–30	28 (31.1%)	62 (68.9%)	2.21 (0.64–5.05)	0.059^*∗*^
31–35	14 (34.1%)	27 (65.9%)	1.93 (0.74–4.97)	0.174^*∗*^
≥36	16 (50.0%)	16 (50.0%)	1	
Ethnicity				
Agnuwa	48 (36.9%)	82 (63.1%)	0.95 (0.60–1.48)	0.809
Nuer	82 (35.6%)	148 (64.4%)	1	
Occupation				
Students	26 (30.6%)	59 (69.4%)	0.82 (0.31–1.59)	0.395
Housewife	51 (38.3%)	82 (61.7%)	1.31 (0.56–1.58)	0.818
Employed	11 (37.9%)	18 (62.1%)	1.20 (0.72–2.33)	0.393
No specified job	42 (37.2%)	71 (62.8%)	1	
Income availability^*∗∗*^				
Yes	32 (43.8%)	41 (56.2%)	1	
No	98 (34.1%)	189 (65.9%)	0.66 (0.39–1.12)	0.225
Educational status				
Illiterate	53 (46.9%)	60 (53.1%)	1.95 (1.23–3.08)	0.004^*∗*^
Literate	77 (31.2%)	170 (68.8%)	1	
Marital status				
Married	103 (35.8%)	185 (64.2%)	1	
Unmarried^*∗∗∗*^	27 (37.5%)	45 (62.5%)	0.93 (0.54–1.58)	0.784
Duration of stay in refugee camp				
<18 months	65 (43.9%)	83 (56.1%)	1.77 (1.14–2.74)	0.010^*∗*^
≥18 months	65 (30.7%)	147 (69.3%)	1	
Trimester				
First	6 (17.6%)	28 (82.4%)	1	
Second	46 (27.9%)	119 (72.1%)	2.43 (1.53–3.85)	0.002^*∗*^
Third	78 (48.4%)	83 (51.6%)	4.38 (1.72–11.16)	<0.001^*∗*^
Gravidity				
Primigravidae	23 (29.1%)	55 (70.5%)	1	
Multigravidae	107 (37.9%)	175 (62.1%)	1.46 (0.85–2.51)	0.170^*∗*^
Parity				
Nulliparous	30 (33.0%)	61 (67.0%)	1	
Primiparae	12 (38.7%)	19 (61.3%)	0.78 (0.33–1.81)	0.562
Multipara	88 (37.0%)	150 (63.0%)	0.84 (0.50–1.39)	0.498
Birth interval in months				
≤24	45 (39.1%)	70 (60.9%)	1.19 (0.70–2.02)	0.506
>24	43 (35.0%)	80 (65.0%)	1	
Blood loss^†^				
Yes	21 (41.2%)	30 (58.8%)	1.28 (0.70–2.35)	0.417
No	109 (35.3%)	200 (64.7%)	1	
Prolonged menstruation^††^				
Yes	19 (46.3%)	22 (53.7%)	1.62 (0.84–3.12)	0.150^*∗*^
No	111 (34.8%)	208 (65.2%)	1	
History of abortion				
Yes	31 (48.4%)	33 (51.6%)	1.87 (1.08–3.23)	0.025^*∗*^
No	99 (45.4%)	119 (54.6%)	1	

COR: crude odds ratio, CI: confidence interval, ^*∗*^ *P* ≤ 0.20, candidates for multivariate logistic regression analysis, 1: reference group, ^*∗∗*^any source of personal income other than aid or donation, ^*∗∗∗*^single, divorced, and widowed, ^†^bleeding (hemorrhage) during index pregnancy, and ^††^history of prolonged menstruating for longer than 7 days.

**Table 3:** Nutrition and clinical characteristics of refugee pregnant women attending ANC clinic at Pugnido ARRA Health Center, Gambela, Western Ethiopia, April 15–June 30/2015.

Variables	Anemia		COR (95% CI)	*P* value
	Yes: *n* (%)	No: *n* (%)		
Freq. of meat eating per week				
At most once	88 (45.8%)	104 (55.2%)	2.54 (1.62–3.98)	<0.001^*∗*^
More than once	42 (25.0%)	126 (75.0%)	1	
Freq. of vegetable eating per week				
At most once	46 (32.6%)	95 (67.4%)	0.78 (0.50–1.21)	0.269
More than once	84 (38.4%)	135 (61.6%)	1	
Freq. of fruit eating per week				
At most once	92 (39.3%)	142 (60.7%)	1.50 (0.94–2.38)	0.085^*∗*^
More than once	38 (30.2%)	88 (69.8%)	1	
Staple food				
Fish	19 (43.2%)	25 (56.8%)	1	
Porridge	84 (33.7%)	165 (66.3%)	1.49 (0.78–2.86)	0.228
Injera	23 (38.3%)	37 (61.7%)	1.22 (0.55–2.70)	0.619
Other^*∗∗*^	4 (57.1%)	3 (42.9%)	0.57 (0.11–2.85)	0.494
Freq. of drinking tea immediately after meal per day				
At least once	72 (59.0%)	50 (41.0%)	4.47 (2.80–7.12)	<0.001^*∗*^
Less than once	58 (24.4%)	180 (75.6%)	1	
Freq. of drinking coffee immediately after meal per day				
At least once	50 (52.6%)	45 (47.4%)	2.57 (1.59–4.15)	<0.001^*∗*^
Less than once	80 (30.2%)	185 (69.8%)	1	
MUAC				
<21 cm	50 (73.5%)	18 (26.5%)	7.36 (4.05–13.37)	<0.001^*∗*^
≥21 cm	80 (27.4%)	212 (72.6%)	1	
Number of ANC visits for current pregnancy				
1	97 (35.1%)	179 (64.9%)	1.20 (0.72–1.97)	0.489
≥2	33 (39.3%)	51 (60.7%)	1	
Taken iron/folate supplement^*∗∗∗*^				
Yes	28 (40.6%)	41 (59.4%)	1	
No	102 (35.1%)	189 (64.9%)	1.26 (0.74–2.16)	0.391
Place of previous delivery				
Health institution	41 (33.3%)	82 (66.7%)	1	
Home	59 (40.4%)	87 (59.6%)	0.74 (0.45–1.21)	0.289
ANC follow-up in previous pregnancy				
Yes	34 (28.6%)	85 (71.4%)	1	
No	66 (44.0%)	84 (56.0%)	0.62 (0.37–0.95)	0.018^*∗*^
History of malaria infection				
Yes	91 (42.3%)	124 (57.7%)	1.99 (1.26–3.15)	0.003^*∗*^
No	39 (26.9%)	106 (73.1%)	1	
Positive for current malaria infection				
Yes	30 (52.6%)	27 (47.4%)	2.25 (1.27–4.00)	0.005^*∗*^
No	100 (33.0%)	203 (67.0%)	1	
Positive for intestinal parasite infestation				
Yes	48 (50.5%)	47 (49.5%)	2.28 (1.41–3.68)	0.001^*∗*^
No	82 (30.9%)	183 (69.1%)	1	

COR: crude odds ratio, CI: confidence interval, ^*∗*^ *P* ≤ 0.20, candidates for multivariate logistic regression analysis, 1: reference group, Freq.: frequency, ^*∗∗*^respondents using bread and packed food as their staple food, ^*∗∗∗*^taken iron/folate supplement during the index pregnancy, MUAC: mid-upper arm circumference, and ANC: antenatal care.

**Table 4:** Independent predictors of anemia among pregnant women attending ANC clinic at Pugnido ARRA Health Center in Gambela, Western Ethiopia, April 15–June 30/2015 (*n* = 360).

Variables	COR (95% CI)	*P* value	AOR (95% CI)	*P* value
Trimester				
First	1		1	
Second	2.43 (1.53–3.85)	0.002	2.06 (0.98–3.86)	0.051
Third	4.38 (1.72–11.16)	<0.001	3.12 (1.16–9.83)	0.014^*∗*^
Intestinal parasite infestation				
Yes	2.28 (1.41–3.68)	0.001	2.17 (1.20–3.91)	0.010^*∗*^
No	1		1	
Freq. of meat eating per week				
At most once	2.54 (1.62–3.98)	<0.001	2.00 (1.11–3.58)	0.020^*∗*^
More than once	1		1	
Freq. of drinking tea immediately after meal				
At least once per day	4.47 (2.80–7.12)	<0.001	3.01 (1.74–5.22)	<0.001^*∗*^
Less than once per day	1		1	
MUAC				
<21 cm	7.36 (4.05–13.37)	<0.001	3.90 (1.94–7.84)	<0.001^*∗*^
≥21 cm	1		1	

AOR: adjusted odds ratio, COR: crud odds ratio, CI: confidence interval, ^*∗*^significant association at *P* < 0.05, 1: reference group, Freq.: frequency, and MUAC: mid-upper arm circumference.
